# A Fast Channel Assignment Scheme for Emergency Handling in Wireless Body Area Networks

**DOI:** 10.3390/s17030477

**Published:** 2017-02-27

**Authors:** Sabita Nepal, Amod Pudasani, Seokjoo Shin

**Affiliations:** Department of Computer Engineering, Chosun University, Gwangju 61452, Korea; sabitanepal2002@gmail.com (S.N.); amod13@gmail.com (A.P.)

**Keywords:** WBAN, emergency handling, MAC frame structure, correlated traffic

## Abstract

Ubiquitous healthcare is a promising technology that has attracted significant attention in recent years; this has led to the realization of wireless body area networks (WBANs). For designing a robust WBAN system, the WBAN has to solve the drawbacks of wireless technology. Also, a WBAN has to support immediate, reliable data transmission for medical services during emergencies. Hence, this study proposes a new MAC superframe structure that can handle emergencies by delivering strongly correlated regular data to a caretaker, within a certain time threshold. Simulation results demonstrate that the proposed MAC protocol achieves low latency and high throughput.

## 1. Introduction

The existence of the wireless sensor network (WSN) paradigm [1] has come along with the development of information and communication technology and the exponential growth of user demand for large-scale communication infrastructure. As a special-purpose sensor network application, the wireless body area network (WBAN) has emerged as a key technology capable of short range, low power, and highly reliable wireless communication. In addition, WBANs can be used externally, in close proximity to a body, or internally, inside an organ of a patient, for the diagnosis of several diseases including critical diseases and for providing services related to real-time health monitoring. Moreover, WBANs consist of different sets of medical sensors capable of sensing and monitoring various medical states. These sensors are equipped with communication boards and antennas used for forwarding the information to a medical system that will display, store, and process the data to detect the presence of any medical anomaly. Today, the WBAN is considered a key technology in providing healthcare services anywhere, anytime, and it is expected to play an important role in enhancing the quality of life, especially for the elderly. For designing a robust WBAN system, fundamental wireless networking issues must be addressed and resolved. 

WBAN technology is one of the rapid advancements of wireless communications designed to operate autonomously and connect various sensor nodes to a wireless network. WBANs provide closer interconnection (2–5 m) with stricter technical requirements such as high reliability, extreme power efficiency, security, and safety for the human body. Unfortunately, more protocol details are not fully disclosed in the current version of the IEEE 802.15.6 standard; therefore, a better approach to design a new WBAN system is based on IEEE 802.15.4 standard, which is a mature protocol that has been applied to many fields.

IEEE 802.15.4, published in 2006, specifies the physical (PHY) and media access control (MAC) layers for short range wireless communications. IEEE 802.15.4 provides a solution for a low rate, low power wireless personal area network (WPAN) in the personal operating space (POS) of 10 m, which is typically a sensor network [2]. The IEEE 802.15.4 standard is widely used for WBANs because of its features such as energy efficiency, scalability, and design flexibility. However, the standard is currently unable to meet all stringent network requirements of a WBAN because IEEE 802.15.4 does not have any emergency handling or traffic differentiation mechanisms. Unawareness of an emergency signal and its correlated traffic in terms of latency may cause a more critical situation.

Traffic generated in the human body can be classified as either irregular emergency traffic that occurs suddenly owing to the unexpected onset of a health condition or regular periodic traffic that continuously occurs before and after irregular emergency traffic. The human body signals are correlated in that one event which can trigger another series of events. When an emergency is triggered, the correlated medical data associated with the emergency event should be reported urgently within the deadline to make an immediate and accurate diagnosis. If some of the correlated medical data fails to be reported within a delay margin, the outcomes may be life threatening or result in serious and lasting damage to organs. 

In [3] the sensors commonly deployed in WBAN system are given. We have listed those sensors in the Table 1 along with their measurement and possible diagnosis. Some of the medical sensors are critically important that can act as an emergency alarm. In [4] the criteria for activation of medical emergency state have been given. For example, an oxygen saturation level (SpO2) less than 90% or cardiac arrest is the health indication that requires immediate emergency service. Thus, during these emergency conditions, the medical data from other sensors like temperature, a respiration rate, etc. is also equally important for a proper diagnosis and better health care service in an emergency situation. In [5] the real time monitoring of electrocardiogram (ECG) through IEEE 802.15.4 is studied. The data rate of one channel ECG signal with 200 Hz and 16-bit sampling resolution is 3.2 kbps. Similarly, in [6] physiological data characteristics with required data rate are given. Here, physiological signal from temperature sensor only requires 0.0024 kb/s. Similarly, blood pressure requires 1.2 kbps. If we sample the SpO2 with 50 Hz and 16-bit sampling resolution, the data rate would be 0.8 kbps. 

Due to the lack of a traffic prioritization mechanism in IEEE 802.15.4, the critical data does not get any prioritized access in the medium. Therefore, emergency data handling is not efficient using its MAC protocol. In this paper, a new WBAN MAC protocol is proposed to minimize the delay associated with regular periodic traffic as well as the correlated traffic in an emergency event. The new MAC frame structure provides an emergency contention period (ECP) to transmit emergency event data that invokes a dedicated transmission slot (DTS) allocation in an inactive period to transmit the correlated traffic with the corresponding emergency event.

The bio medical sensors are usually used with high risk patients and the quality of service should be assured. The proposed method is especially designed for WBAN by modifying the IEEE 802.15.4 MAC to support short range, low power and reliable wireless communication in vicinity or inside of a living tissue. The proposed MAC could be developed as a medical system with a scheme design, its software realization and the hardware implementation. In this WBAN application, the sensors implanted in/on the human body will be able to transfer the physiological measurements to a control unit and then to all the remote stations in a network. 

The remainder of this paper is organized as follows: Section 2 gives an overview of IEEE 802.15.4. Section 3 briefly describes the proposed super frame structure, and the principle operation of the proposed scheme is described in Section 4. Numerical analysis and simulation results are discussed in Section 5 and Section 6, respectively. Finally, Section 7 concludes the paper.

## 2. Related Works

A BAN is a collection of sensor nodes implanted or attached to a human body capable of communication. BANs are a promising technology which can drastically reform the variety of medical applications. A comprehensive study of BANs has been highlighted in [7] where the authors delve into overall BAN systems by better describing the state of art technologies. A detailed description of the sensor devices, aspects of BANs at the physical layer and data link layer, and the investigation of radio technology for BANs is outlined in the manuscript. 

There are plenty of challenging issues in BANs that limit their wide implementation. One of the challenges prevailing in BANs is a lack of high level software abstraction which results in the programming complexity of the system. In [8] an open source programming framework named as signal processing in node environment (SPINE) is presented which is designed to support rapid, flexible prototyping and management of sensor applications, and to address the requirement of a BAN framework with proper abstraction of layers. The two software architectures of SPINE; SPINE node and SPINE coordinator provide an application program interface (API) to manage BAN applications. SPINE maximizes the effectiveness of applications development in BSN and could be utilized for a rapid development of different BAN applications. The performance analysis of SPINE carried out for the most common hardware/software sensor platforms has been found to efficiently address the identified requirements for a BAN framework. 

In order to achieve high throughput in an energy efficient manner, the design of an efficient MAC protocol is essential. Time synchronized channel hopping (TSCH) is currently the most efficient solution for collision free, interference avoiding communication. For maintaining the time-frequency slotting mechanism, a centralized coordinator is required. In [9], a decentralized time synchronized channel swapping (DT-SCS) MAC for ad-hoc wireless networks has been proposed. The proposed MAC protocol leverages pulsed-couple oscillators that simultaneously carry out the synchronization and desynchronization over multiple channels where the nodes randomly join a channel and automatically spread across the available channels. The delay occurred due to the channel switching mechanism is a challenge for its implementation in WBAN with a group of nodes in the same channel. 

The use of machine learning for improvement in MAC performance is presented in [10]. A reinforcement learning algorithm has been used for management of an adaptive radio scheduling. The proposed QL-based MAC protocol allows each node to determine better scheduling policy by learning the neighbor behavior and adapting the changes in the local traffic conditions. The performance of QL-MAC has been compared with the conventional MAC protocols. The results show that the adaptive behavior of QL-MAC guarantees better network performances with respect to both the packet delivery ratio and the average energy consumption.

Similarly, a number of MAC protocols have been heavily researched and proposed based on IEEE 802.15.4. As WBAN technology has gained worldwide interest, considerable research efforts have been dedicated to proposing new MAC protocols based on 802.15.4 in order to satisfy the stringent requirements of WBANs.

In [11], the authors presented a priority guaranteed MAC protocol, in which the data and the control channels are separated to support collision-free high data rate communication. Application specific control channels are adopted to provide priority guarantee to the life-critical medical applications over the much busier consumer electronics (CE) traffic. Improvements in throughput and energy efficiency are achieved from the given MAC protocol. 

Kim et al. [12] proposed a mechanism for IEEE 802.15.4 to provide a traffic differentiation scheme based on the contention window (CW) size and backoff exponent (BE). In this scheme, higher-priority-class nodes have lower CW and BE values than the others. CW has more effect on the saturation throughput, while BE affects the average delay of every device. Therefore, the tuning of the data latency could be performed by varying the BE, and better throughput could be achieved by adjusting the CW size. 

Kwak and Ullah [13] have proposed a traffic-adaptive MAC for handling emergency and on-demand traffic, in which a table is maintained to store the traffic patterns of the nodes. The authors modified the MAC superframe structure to include the configurable contention access period (CCAP); however, the rest of the superframe parts resemble the conventional IEEE 802.15.4 MAC. This superframe helps to solve the idle listening and overhearing problems by exploiting the traffic information of the nodes.

Kim et al. [14] focused on emergency handling schemes for WBANs. They proposed a superframe structure with mixed period (MP) and extended period (EP). In MP, the contention access period (CAP) slot, also called the contention time slot (CTS), is inserted in front of the guaranteed time slot (GTS) for immediate transmission of emergency data, while EP consists of an extending request period (ERP), a reallocated contention-free period (CFP), and an additional CAP. EP guarantees transmissions of a failed slot in MP at the reallocated CFP. The authors of the paper claim that MP and EP can handle emergency data with low latency.

Lee et al. [15] have proposed an enhanced MAC protocol of IEEE 802.15.4 for health-monitoring applications with an enhanced superframe structure containing a polling period (PP) and an emergency slot (ES) for emergency handling. ES is a quite short period where data transmission is described by success or failure. The protocol contains a long CFP followed by an inactive period.

Ranjit et al. [16] have also proposed an emergency-handling MAC protocol for health-monitoring applications using an ERP period and emergency beacon. This superframe handles the emergency traffic by minimizing the delay with a speedy channel assignment for the emergency user.

The PNP-MAC protocol [17] is also based on the IEEE 802.15.4 superframe structure. It can flexibly handle applications with diverse requirements through fast, preemptive slot allocation, non-preemptive transmission, and superframe adjustments. This MAC inherits the benefits of contention-based and contention-free medium access techniques, hence supporting various types of traffic, including continuous streaming, periodic data, time-critical emergency alarm, and non-periodic data. It supports QoS in accordance with the priority of traffic.

The authors in [18] proposed an opportunistic contention decision period (OCDP) MAC protocol for contention-based medical and CE applications. To support bursty CE data and emergency medical data, the proposed WBAN MAC protocol provides a temporary switching method between the inactive period and the opportunity period through OCDP and a four-mode opportunity period. 

In [19], the authors proposed a Medical MAC (MedMAC) protocol for WBANs to improve channel access mechanisms and reduce energy dissipation. This protocol includes contention-free channel access over a variable number of TDMA channels, energy efficient and dynamically adjustable time slots, a novel adaptive and low-overhead TDMA synchronization mechanism, optimized energy efficiency by dynamically adjusting the QoS requirements using ongoing traffic analysis, and optional contention period used for low grade data, emergency operation, and network initialization procedures.

Most of the previous studies have not been successful enough to address the fast delivery of real-time emergency-related human life-critical data to the coordinator within a limited time requirement. The main cause of the long latency is the lack of interaction between nodes and a coordinator in the active period as well as the relatively long inactive period designed to save energy. Therefore, in this paper, a new frame structure and its operation are proposed for fast channel allocation for the sake of all emergency-related vital data transmissions.

## 3. IEEE 802.15.4 MAC Overview

**Figure 1:**
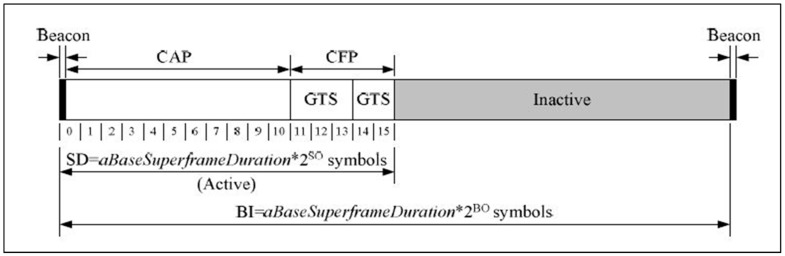
IEEE 802.15.4 superframe structure.

IEEE 802.15.4 has become a very popular technical standard designed especially for low power and low data rate applications [2]. The IEEE 802.15.4 MAC can operate in two modes: beacon-enabled and non-beacon-enabled mode. In the beacon-enabled mode, the network is controlled by a coordinator that regularly transmits beacons with information such as device synchronization and association control. The period between two consecutive beacons is called a superframe, which is illustrated in Figure 1. 

A superframe is always bordered by the two successive beacons and consists of an active period and an inactive period. The length of an entire superframe is defined as the beacon interval (BI), and the length of the active part of the superframe, or superframe duration (SD), are defined as:

$$\begin{array}{c} \mathrm{BI}=\mathrm{aBaseSuperframeDuration}*2^{\mathrm{BO}}\\ \mathrm{SD}=\mathrm{aBaseSuperframeDuration}*2^{\mathrm{SO}}\\ \text{Inactive Period}=\mathrm{BI}-\mathrm{SD} \end{array}$$

where 0 ≤ SO ≤ BO ≤ 14, aBaseSuperframeDuration = 960 symbols, BO = Beacon Order, and SO = Superframe Order. 

The active period of the superframe is divided into 16 equally sized time slots for data transmission. The active period is further divided into two components: a CAP and a CFP. The CAP immediately follows the superframe beacon and lasts until the CFP begins. The channel access mechanism during CAP is a slotted CSMA/CA mechanism. A transmission in the CAP needs to be completed before the end of the CAP. If this is not possible, it defers its transmission until the CAP of the next upcoming superframe. CFP starts immediately after the end of the CAP and should be completed before the start of the next beacon frame. Unlike CAP, channel access in CFP is based on reservations and is free of contention. All GTSs are allocated as per request by the PAN coordinator if there are available resources. The coordinator allocates up to seven GTSs that may contain one or more time slots either in the transmitting or receiving direction. The allocation of the GTS cannot reduce the length of the CAP to less than aMinCAPLength (440 symbols). In addition, it is noteworthy that a device to which a GTS has been allocated can also transmit during the CAP.

## 4. Proposed MAC Protocol

**Figure 2:**
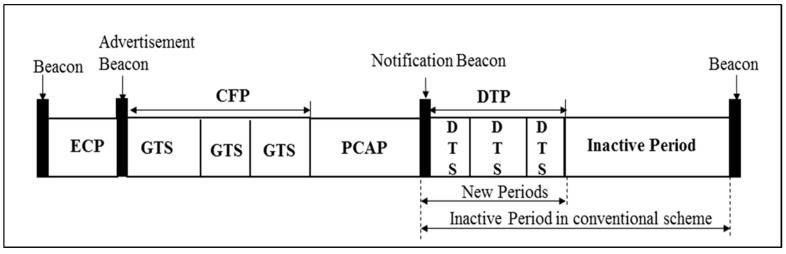
Modified Superframe Structure in the Proposed Protocol.

In the conventional beacon-enabled IEEE 802.15.4 MAC, after CAP and CFP, the sensor nodes and a network coordinator (NC) enter into sleep mode during an inactive period. In our proposed scheme, this inactive period is partly utilized for sending strongly correlated medical data only when an emergency condition is noted. The modified frame format consists of the beacon, emergency contention period (ECP), advertisement beacon (AB), contention-free period (CFP), periodic contention access period (PCAP), notification beacon (NB), data transmission period (DTP), and inactive period, which is shown in Figure 2. Note that the AB and NB are not a normal type of beacon, but simple responses of the requests from the nodes to a NC.

The superframe starts with a beacon period, where the beacon contains the information on the addressing fields, the superframe specification, the GTS fields, the pending address fields, and other PAN related information. 

The ECP starts just after the beacon and ends before the beginning of the AB. In this period, if any node has emergency data to be reported, the node tries to transmit the data directly to the NC by using ECP period. The usage of ECP is equal to that of CAP, so that all the functional, procedural behavior of nodes in ECP is equal to those in CAP. Upon receiving a valid emergency event data from sensor nodes in the ECP, the NC broadcasts a set flag through the AB to indicate the presence of emergency event to all nodes. If there is no emergency, a reset flag is broadcasted through the AB.

CFP functions in the modified superframe structure are same as those of the conventional IEEE 802.15.4 standard. The PCAP starts immediately after the CFP. Nodes with regular periodic data are allowed to report and transmit in this period. If nodes receive the set flag through the AB, then they send a DTS request frame (DTS-RF) to a NC in order to transmit correlated medical data invoked by an emergency data transmission. Otherwise, they send a GTS request frame to a NC in order to operate normally as in IEEE 802.15.4. CSMA/CA is also used in the PCAP.

The NB follows the PCAP, whose usage is broadcast by a coordinator only if any DTS-RF is requested in the PCAP. Otherwise, the NB and DTP periods belong to an inactive period. The NB contains the transmission schedule (i.e., the allocation of DTS). All nodes who have issued a DTS-RF in the PCAP should listen to the NB to check whether their request in the PCAP is acknowledged by the coordinator. 

The DTP is a TDMA-based contention-free access period that appears after the NB. The DTP is divided into a number of slots called DTSs. The length of a DTS is determined to accommodate regular periodic data and an ACK message. The maximum number of such DTSs allocated for an instance in a superframe is equal to *M*. The value of *M* could be adjusted according to the density of correlated medical traffic in the network. The maximum number of allowed GTSs in the conventional IEEE 802.15.4 MAC protocol is 7. In our proposed scheme, we also considered *M* equal to 7 in order to maintain consistency with the conventional IEEE 802.15.4 superframe structure. It is noteworthy that the performance of the proposed scheme is a function of resources allocated to the emergency handling nodes and the regular periodic data nodes, which are the size of ECP and *M*, respectively. Generally, any changes to those parameters will lead to performance variation. However, for the purpose of our comparative analysis, we have exploited the given resource block in the conventional superframe structure to a maximum possible limit and compared it with the conventional structure.

Let us assume that a node has emergency data to transmit, and some other nodes have its follow-up correlated regular data advanced to the ECP in a superframe. The emergency data can be sent to a NC through the very next ECP period. Now, since strongly correlated medical data has its importance in the event of an emergency, it also needs to be reported to a NC within the delay margin. When a NC receives the emergency data in ECP, it then broadcasts a set flag through the AB to all nodes to inform them that the emergency has occurred, so that strongly correlated medical traffics can be sent.

**Figure 3:**
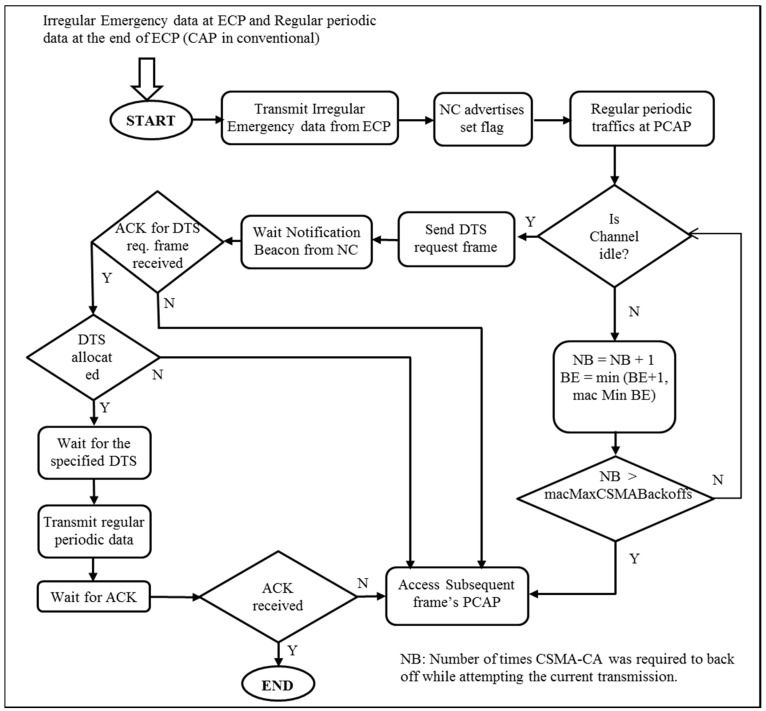
Flow Diagram of proposed scheme.

Nodes with mundane regular data wait until the start of the PCAP and perform random access governed by the CSMA/CA protocol. Once the channel is idle, the node sends a DTS-RF and waits for an ACK. If the node receives an ACK for the request and DTS slot allocation information (regarding which DTS has been assigned to whom) by a coordinator through the NB, it transmits its regular data in the DTS allocated to it. On the other hand, if the node does not receive an ACK or a DTS assignment in the NB, the node waits until the next PCAP for data transmission by contention. The set of procedures operated in the proposed scheme is summarized in Figure 3 as a flow diagram. 

## 5. Numerical Analysis

### 5.1. Performance Metrics

In this section, we formulate some expressions to calculate three important performance measures including minimum achievable delay, maximum achievable throughput, and maximum energy consumption. These performance measures are achieved in ideal channel conditions such that there is no packet failure in the network either due to collision or channel error. Even though the ideal channel condition is considered in this analysis, the results can be used as a reference to show the patterns of the proposed scheme. 

#### 5.1.1. Delay

For sending a packet of size *x*, the minimum achievable delay for the conventional IEEE 802.15.4 can be calculated using the following expression:

(1) $$D_{c}\left(x\right)=T_{\mathrm{CFP}}+T_{\mathrm{Inactive}}+T_{\mathrm{Beacon}}+T_{\mathrm{SIFS}}+T_{\mathrm{BO}}+T\left(x\right)+T_{\mathrm{TA}}+T_{\mathrm{ACK}}+T_{\mathrm{IFS}}\left(x\right)$$

where T_CFP_ is the duration of the CFP, T_Inactive_ is the duration of the inactive period, T_Beacon_ is the beacon duration, T(x) is the time taken to transmit a packet of size *x*, T_TA_ is the turnaround time, T_ACK_ is the time taken for ACK transmission, T_IFS_(x) is the IFS time for a packet of size *x*, and T_BO_ is the duration of the backoff period. Note that in IEEE 802.15.4, T_IFS_(x) is 640 μs for *x* greater than 18 bytes, otherwise it is 192 μs. During the backoff procedure in beacon-enabled IEEE 802.15.4, a node performs a clear channel assessment (CCA) twice. Therefore, backoff duration can be expressed as:

(2) $$T_{\mathrm{BO}}={\mathrm{BO}}_{\mathrm{slots}}*T_{{\mathrm{BO}}_{\mathrm{slot}}}+2T_{\mathrm{CCA}}$$

where BO_slots_ is the average number of slots in which the node deferred its transmission, T_BOslot_ is the slot duration (320 μs), and T_CCA_ is the time required for CCA.

**Figure 4:**
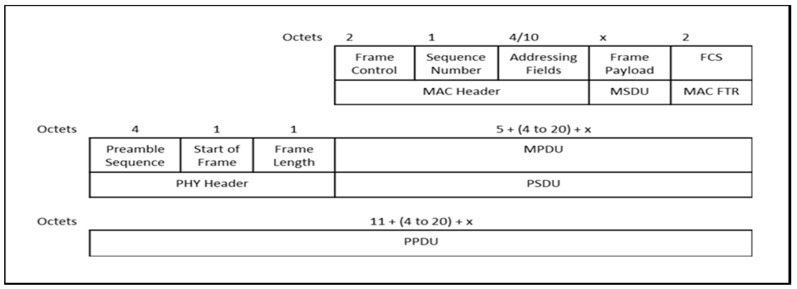
Frame format (IEEE 802.15.4).

Based on the frame format shown in Figure 4, we calculate T(x) to be:

(3) $$T\left(x\right)=8*\frac{L_{\mathrm{PHY}}+L_{\mathrm{MAC}\_\mathrm{HDR}}+L_{\mathrm{address}}+x+L_{\mathrm{MAC}\_\mathrm{FTR}}}{R_{\mathrm{data}}}$$

where L_PHY_ is the length of the PHY header in bytes, L_MAC_HDR_ is the length of the MAC header in bytes, L_address_ is the length of the MAC address, L_MAC_FTR_ is the length of the MAC footer in bytes, and R_data_ is raw data rate in bits per second (bps). Finally, T_ACK_ can be calculated as:

(4) $$T_{\mathrm{ACK}}=8*\frac{L_{\mathrm{PHY}}+L_{\mathrm{MAC}\_\mathrm{HDR}}+L_{\mathrm{MAC}\_\mathrm{FTR}}}{R_{\mathrm{data}}}$$

Similar to the conventional scheme, we calculate the minimum achievable delay for the proposed scheme by adding the required duration for packet exchange and protocol timing specifications. The minimum achievable delay of the proposed scheme for reporting the medical data in the case of an emergency event using an *x*-byte packet is:

(5) $$D_{P}\left(x\right)=T_{\mathrm{CFP}}+T_{\mathrm{AB}}+T_{\mathrm{SIFS}}+T_{\mathrm{PCAP}}+T_{\mathrm{SIFS}}+T_{\mathrm{NB}}+T_{\mathrm{SIFS}}+T\left(x\right)+T_{\mathrm{TA}}+T_{\mathrm{ACK}}+T_{\mathrm{IFS}}\left(x\right)$$

where T_AB_ is the AB duration, T_PCAP_ is the PCAP duration, and T_NB_ is the NB duration.

Note that the length of the CFP is determined from the number of GTSs allocated by the coordinator. At a maximum, seven such GTSs can be allocated in a time provided when there are sufficient resources available. Each GTS can occupy a single or more transmission slots. Therefore, the smallest length of CFP denoted by CFP_min_ is a single GTS extending to a single transmission slot. On the basis of these varying CFP lengths, different cases of the delay calculation for the proposed scheme are calculated for best case delay and worst case delay.

#### 5.1.2. Throughput

Once the delay for the conventional scheme and the proposed scheme are calculated, the maximum achievable throughput for both the schemes can be calculated as:

(6) $${\mathrm{Th}}_{c}\left(x\right)=8*\frac{x}{D_{c}\left(x\right)}$$

(7) $${\mathrm{Th}}_{P}\left(x\right)=8*\frac{x}{D_{P}\left(x\right)}$$

where Th_c_ and Th_p_ are the throughputs for the conventional scheme and the proposed scheme, respectively.

#### 5.1.3. Energy Consumption

The maximum energy consumption can be obtained from the above throughput and delay, which is given as:

(8) $$E_{c}\left(x\right)=2P_{r}T_{\mathrm{Beacon}}+P_{i}T_{\mathrm{BO}}+P_{t}T\left(x\right)+P_{r}T_{\mathrm{ACK}}$$

(9) $$E_{p}\left(x\right)=3P_{r}T_{\mathrm{Beacon}}+P_{i}T_{\mathrm{BO}}+P_{t}T\left(x\right)+P_{r}T_{\mathrm{ACK}}$$

where E_c_ and E_p_ are the energy consumptions for the conventional and the proposed scheme, respectively; P_t_, P_r_, and P_i_ are the power of radio transmission, radio reception, and idle listening, respectively; T_Beacon_ is the beacon duration, T_BO_ is the duration of the backoff period, and T(x) is the time taken to transmit a packet of size x; and T_ACK_ is the time taken for an ACK transmission.

### 5.2. Numerical Results

In this section, we have compared the delay, throughput, and energy consumption of our proposed scheme with the conventional scheme using the expressions derived in Section 5.1 and considering the parameters in Table 2. 

The parameters in the table are taken from an IEEE standard [2,20]. In our numerical analysis, medical events are considered to occur at the beginning of the CFP. Two different cases for delay and throughput, namely the best case and worst case, are presented and discussed for both the proposed and the conventional scheme.

Table 3 and Table 4 show the minimum achievable delay of the proposed scheme and the conventional IEEE 802.15.4 for the best and worst cases, respectively. The values in the table are calculated from Equation (5) in Section 5.1.1. It is observed from Table 3 and Table 4 that for different packet sizes, the delay of the proposed scheme is less than that of the conventional IEEE 802.15.4. It can be also seen that the delay is not greatly affected by the packet size. From the table, it is clear that the proposed scheme satisfies the delay requirement of WBANs stated in [20] which is 125 ms for real time medical application such as tele surgery, while the conventional scheme does not. Note that since the healthcare monitoring application targeted in our study is not real time medical application, the delay requirement of 125 ms is not used as an upper limit but a guide line of the performance (there are no explicit delay requirements of emergency reporting yet). The comparison in the table is very much convincing to show the proposed method is better in terms of delay compared with conventional IEEE 802.15.4 when delivering correlated medical traffic in case of emergency events. Consequently, the proposed scheme is more suitable than the conventional scheme for handling emergency data in delay-sensitive WBAN applications.

**Figure 5:**
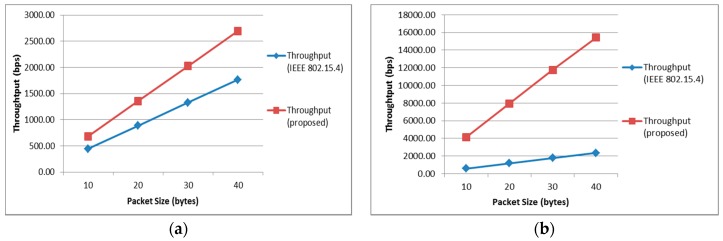
Maximum achievable throughput showing the best and worst cases of the proposed and conventional schemes according to the packet size: (**a**) Best Case; (**b**) Worst Case.

Figure 5 shows that the maximum achievable throughput of the proposed scheme goes up linearly with respect to the packet size. Moreover, throughputs of the proposed scheme are higher than those of the conventional scheme in both cases.

**Figure 6:**
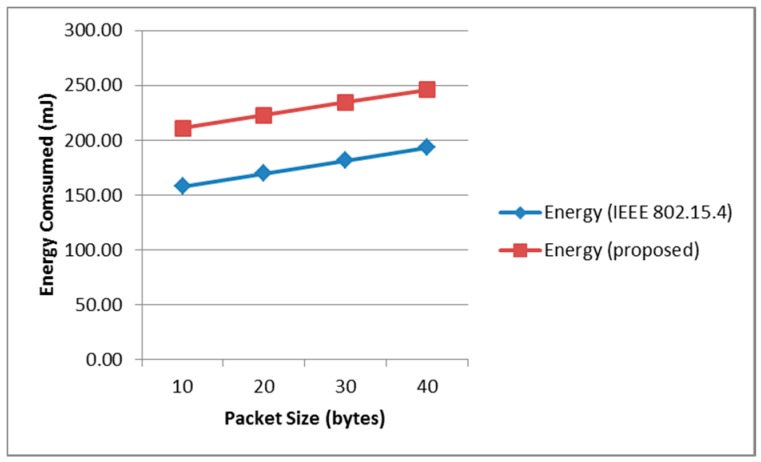
Maximum Energy Consumption of the proposed scheme and the conventional IEEE 802.15.4.

Figure 6 shows the maximum energy consumption according to the packet size for the proposed and conventional protocols. The proposed scheme consumes more energy than the conventional scheme. From the comparisons, we can be assured that the proposed scheme shows better performance in terms of delay and throughput than that of the conventional scheme. Moreover, the proposed scheme, at the expense of more power consumption, can deliver emergency data and its correlated regular periodic data within a much shorter time than that of the standard protocol, IEEE 802.15.4.

## 6. Simulation Results and Discussion

To evaluate the proposed scheme, Castalia-3.2 [21], a network simulator specifically designed for sensor and body area networks based on OMNeT++ platform [22], has been used. Castalia inherits experimentally measured signal attenuation values from real world scenarios that define a map of path loss. The model is not simply the connections between nodes but a complex model for temporal variation of path loss from mobility of nodes and interference based on received signal strength. The physical layer parameters are defined according to the IEEE 802.15.4 standard. For our proposed scheme, we have modified the conventional IEEE 802.15.4 MAC protocol in the Castalia simulator including the modification of the superframe structure and triggering mechanism of the emergency event. The simulation in this paper was carried out in a star topology, with single-hop communication between the NC and sensor nodes. The sensor nodes are randomly deployed within an area of 4 m radius. Poisson distribution with varying mean inter-arrival times (T_mean_) has been used for inter-arrival process of packets. All the nodes intend to transmit the first packet randomly during the contention access period. The number of nodes is varied from 5 to 60 biomedical sensor nodes increased by 5, and the packet length is fixed to 40 bytes. The 40-bytes packet size as a medical traffic has been proved to show lower end to end latency and an acceptable packet delivery rate in [23]. Considering the medical ECG application with 2 lead ECG nodes, the data rate will be 6.4 kbps [5], which will be 800 bytes in a second. Thus, our assumption of 40 bytes of packet size can accommodate this medical data in the modified superframe structure. The types of traffic from the sensor nodes are classified as emergency event traffic and strongly correlated traffic of an emergency event. During the simulation period, out of total traffics generated by all the nodes, 10% is assumed to be emergency event traffic while 90% is strongly correlated traffic. Note that the detailed simulation parameters and their values are summarized in Table 5 (some parameters are adopted from reference [24]). 

The simulation results of the proposed scheme are evaluated against the conventional IEEE 802.15.4. In the comparison, end-to-end delay and throughput are considered as the performance measures. Note that the simulation has been performed to get the results for correlated traffic and regular traffic when an emergency event occurs, respectively. 

### 6.1. End-to-End Average Packet Delay

**Figure 7:**
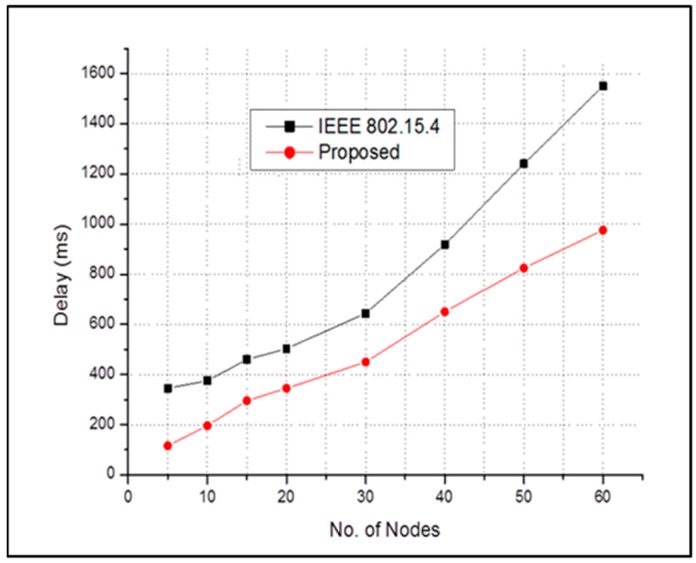
End-to-end average packet delay of all sensor nodes in case of emergency event.

Figure 7 shows the end-to-end average packet delay performance for both schemes in the presence of emergency events. Note that 10% of the total traffic generated in the network is regarded as the emergency event traffic. The results compares the proposed scheme and conventional IEEE 802.15.4. Whenever the channel becomes busy, the sensor nodes have to back off more often, which increases the delay. In general, the delay for both schemes increases as the number of nodes increases. If a greater number of nodes are added to the network, then there is a sharp increase in delay. For every observation point, the proposed scheme has a lower delay than that of the conventional one. In an emergency event, the correlated traffics are delivered utilizing the inactive period which reduce the delay of the proposed scheme while conventional IEEE 802.15.4 requires subsequent frames to deliver the packets.

**Figure 8:**
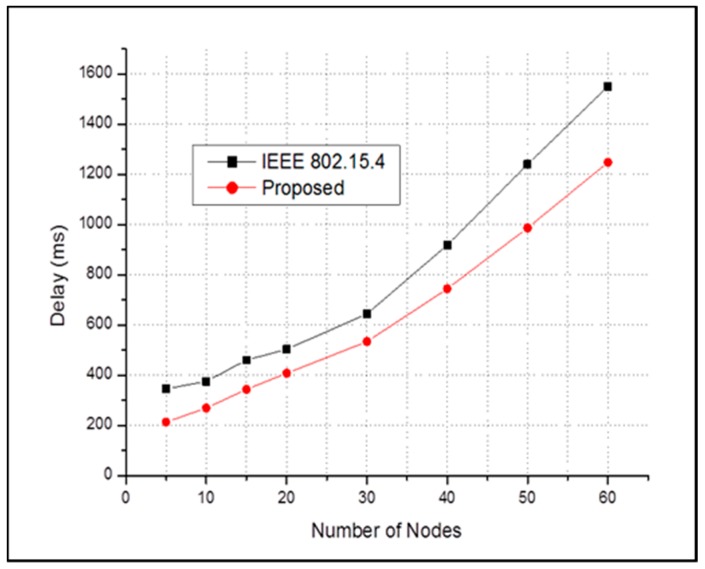
End-to-end average packet delay of all sensor nodes in case of **NO** emergency event.

Similarly, Figure 8 shows the end-to-end delay performance of the regular periodic traffics for both schemes in the absence of emergency event. It is clear that our proposed MAC protocol for emergency handling performs better with respect to delay performance than the conventional IEEE 802.15.4 when it is not emergent. 

### 6.2. End-to-End Average Throughput

**Figure 9:**
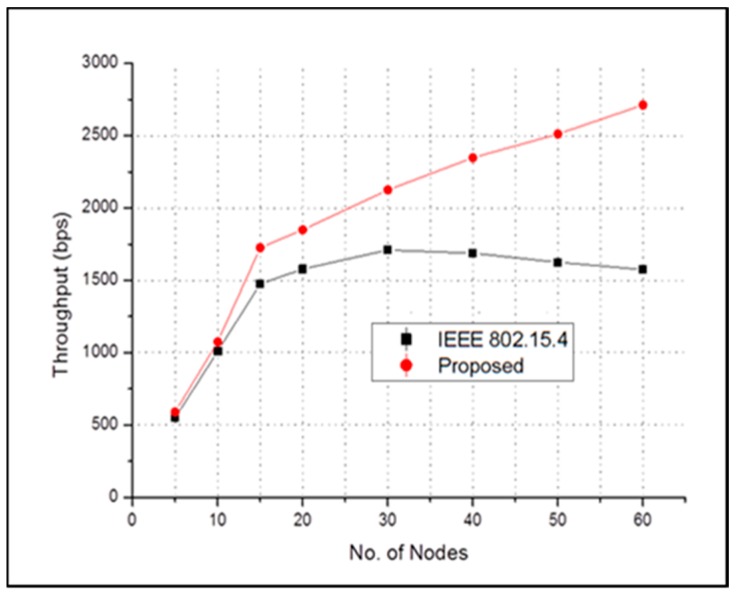
End-to-end average throughput in case of emergency event.

Figure 9 shows the end-to-end average throughput of the proposed scheme and the conventional IEEE 802.15.4. Here, throughput increases as the number of nodes increases up to a certain point for both schemes. However, the proposed scheme has higher throughput than the conventional IEEE 802.15.4. In addition, after a certain point, throughput tends to be saturated. This is because the system can only accommodate a certain number of nodes, and, as the number of nodes increases to the network, the probability of collision also increases. 

**Figure 10:**
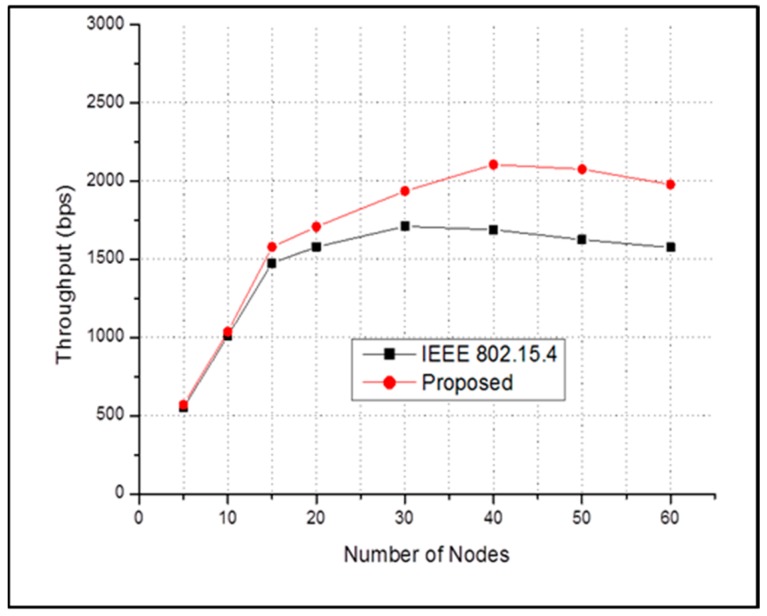
End-to-end average throughput in case of **NO** emergency event.

Figure 10 shows the throughput versus the number of nodes for both schemes when there is no emergency event. From the figure, it can be observed that if there are no emergency event, our proposed MAC protocol has higher throughput than conventional IEEE 802.15.4.

## 7. Conclusions

Medical data from biosensors in or on the human body is highly correlated with each other. When an emergency event is triggered, the delivery of this correlated medical traffic is even more urgent. In this paper, we proposed a modified IEEE 802.15.4 MAC protocol that opportunistically utilizes the inactive period for handling emergency events. In our modified superframe structure, when an emergency alarm is triggered, the strongly correlated medical data is sent through the inactive period. The DTS frame in the inactive period is utilized to transmit the correlated medical data when an emergency event is occurred in the network. The use of inactive period in our mechanism will thus offer a transmission of strongly correlated data within a minimum latency. 

We carried out a numerical analysis and a simulation for the proposed scheme. We also compared our proposed protocol with the conventional IEEE 802.15.4. The numerical results indicate a lower delay requirement, better throughput performance and high energy consumption offered by the proposed scheme. Similarly, from the simulation results, proposed scheme is found to have lower delay and higher throughput for both emergency events as well as non-emergency events. The use of inactive period allows reduction in the delivery time when the correlated medical data is transferred in an emergency event. This study concludes that the proposed scheme can be easily designed with modification of the IEEE 802.15.4 MAC protocol for efficient handling of emergency events by delivering the correlated medical data and an emergency data simultaneously with a minimum delay.

## Figures and Tables

**Table 1:** Commonly employed WBAN sensors with their measurement and possible diagnosis.

WBAN Sensors	Measurement	Diagnosis
Accelerometer	Human Speed	Supplementary Data
Gyroscope	Orientation	Supplementary Data
ECG	Heart Function	Cardiac Attack
Electroencephalogram (EEG)	Brain activity	Epilepsy
Electromyography (EMG)	Muscle Function	Disorder of Nerves, Muscle
Pulse oximetry	Saturation level (SpO2)	Supplementary data
Respiration	Respiration rate	Loss of Consciousness
Blood Pressure	Human body pressure	Hypertension
Blood Glucose	Blood Sugar	Diabetes
Humidity	Humidity	Supplementary Data
Temperature	Body Temperature	Supplementary Data

**Table 2:** System parameters and values.

Parameters/Variables	Values
Data rate (R_data_)	250 kbps
aBaseSuperframeDuration	960 symbols (15.36 ms)
macBeaconOrder (BO)	4
macSuperframeOrder (SO)	3
Beacon Interval (BI)	245.76 ms
aNumSuperframeSlots	16
UnitBackoff Period	20 symbol (0.32 ms)
aMaxSIFSFrameSize	18 octets
aTurnaroundTime	12 symbols (0.196 ms)
macAckWaitDuration	54 symbols (0.864 ms)
Notification Beacon	40 bytes
Beacon	40 bytes (1.28 ms)
macMinBE	3
macMaxBE	5
macMaxCSMABackoffs	4
macMaxFrameRetries	3
CCA	8 symbols
SIFS	12 symbols (0.192 ms)
LIFS	40 symbols (0.64 ms)
Advertisement Beacon	40 bytes
TTA (Turnaround Time)	12 symbols (192 µs)
TIFS(x)	12 symbols (192 µs) (for *x* ≤ 18 Bytes),
	40 symbols (640 µs) (for *x* > 18 Bytes)
LPHY	6 bytes
LMAC_HDR	3 bytes
Laddress	2 bytes
LMAC_FTR	2 bytes
Pt = Power of radio transmission	36.5 mW
P_r_ = Power of radio reception	41.4 mW
P_i_ = Power of idle listening	41.4 mW
Maximum number of DTS (M)	7

**Table 3:** Minimum achievable delay of co-related medical traffic in case of emergency events for the proposed and conventional schemes according to the packet size (best case).

Packet Size	Delay (IEEE 802.15.4)	Delay (Proposed)
10 Bytes	133.76 ms	19.328 ms
20 Bytes	134.528 ms	20.096 ms
30 Bytes	134.848 ms	20.416 ms
40 Bytes	135.168 ms	20.736 ms

**Table 4:** Minimum achievable delay of co-related medical traffic in case of emergency events for the proposed and conventional schemes according to the packet size (worst case).

Packet Size	Delay (IEEE 802.15.4)	Delay (Proposed)
10 Bytes	179.84 ms	117.248 ms
20 Bytes	180.608 ms	118.016 ms
30 Bytes	180.928 ms	118.336 ms
40 Bytes	181.248 ms	118.656 ms

**Table 5:** Simulation parameters.

Parameters/Variables	Values
Data rate (R_data_)	250 kbps
Simulation time	50 s
Frequency band	2.4 GHz
aBaseSuperframeDuration	960 symbols (15.36 ms)
macBeaconOrder (BO)	4
macSuperframeOrder (SO)	3
Beacon Interval (BI)	245.76 ms
aNumSuperframeSlots	16
UnitBackoff Period	20 symbol (0.32 ms)
aMaxSIFSFrameSize	18 octets
Notification Beacon	40 bytes
Beacon	40 bytes (1.28 ms)
macMinBE	3
macMaxBE	5
macMaxCSMABackoffs	4
macMaxFrameRetries	3
CCA	8 symbols
Advertisement Beacon	40 bytes
